# Novel spotted fever group rickettsiae in *Haemaphysalis qinghaiensis* ticks from Gansu, Northwest China

**DOI:** 10.1186/s13071-016-1423-7

**Published:** 2016-03-12

**Authors:** Jifei Yang, Zhancheng Tian, Zhijie Liu, Qingli Niu, Rong Han, Youquan Li, Guiquan Guan, Junlong Liu, Guangyuan Liu, Jianxun Luo, Hong Yin

**Affiliations:** State Key Laboratory of Veterinary Etiological Biology, Key Laboratory of Veterinary Parasitology of Gansu Province, Lanzhou Veterinary Research Institute, Chinese Academy of Agricultural Science, Xujiaping 1, Lanzhou, Gansu 730046 P. R. China; Jiangsu Co-innovation Center for Prevention and Control of Important Animal Infectious Diseases and Zoonoses, Yangzhou, 225009 P. R. China

**Keywords:** *gltA* gene, *ompA* gene, Tick, *Rickettsia* sp, SFG rickettsia

## Abstract

**Background:**

*Rickettsia* spp. are obligate intracellular bacteria and well known as transmitted by arthropods. These pathogens have a broad geographic distribution and a high degree of biological and clinical diversity. This study was conducted to determine the prevalence and molecular characterization of *Rickettsia* spp. in ticks collected from Gansu, where *Borrelia burgdorferi sensu lato* and *Anaplasma phagocytophilum* were previously reported in ticks and ruminants.

**Methods:**

A total of 1,583 questing *Haemaphysalis qinghaiensis* ticks were collected and tested for the presence of *Rickettsia* spp. *gltA* gene by PCR. Samples positive for *gltA* were examined by specific primers targeted for the *ompA* gene of SFG rickettsiae. The infections were further validated by sequencing and positive samples were genetically characterized based on the *gltA* and *ompA* genes.

**Results:**

In total, *Rickettsia* spp. infection was found in 179 (18.5 %) *H. qinghaiensis* tick pools by using PCR and primers specific for the *gltA* gene. Of those, 157 (16.3 %) tick pools were positive for SFG rickettsiae by PCR based on *ompA* gene. Amplification and molecular analysis of the nucleotide sequences of *gltA* and *ompA* genes indicated three potential novel spotted fever group rickettsiae in *H. qinghaiensis* ticks. These three potential novel spotted fever group rickettsiae were clustered together in a subgroup, which represents a sister taxon to and separates from other known four SFG rickettsiae subgroups.

**Conclusions:**

This study revealed a high infection rate of SFG rickettsiae in *H. qinghaiensis* ticks in northwest China. Three potential novel spotted fever group rickettsiae classified into a novel SFG rickettsiae subgroup were identified and named “*Candidatus* Rickettsia gannanii” related strains in recognition of the location where it was first detected.

## Background

Rickettsiae are highly specialized obligate intracellular gram-negative microorganisms that can cause disease in human and/or in other vertebrate and invertebrate hosts with diverse clinical presentations, from asymptomatic to severe [1]. Recently, rickettsioses have been viewed as emerging or reemerging diseases with an almost worldwide distribution [2, 3]. *Rickettsia* was shown to be a large genus encompassing at least 30 recognized species, 19 of which were considered to be human pathogens [4, 5]. A number of other putative species of *Rickettsia* have also been reported on the basis of phylogenetic analyses of different gene loci [5–8]. *Rickettsia* spp. are usually associated with arthropods, and ticks, louse, mites and fleas had been recognized as competent vectors of rickettsial agents [9–12]. Recently, mosquitoes were considered as potential vectors for *R. felis* [13]. In addition, numerous *Rickettsia* spp. or specific DNA have been detected in arthropods other than the aforementioned vectors [2], such as booklice, true bugs, white fly, etc.

Historically, most of the recently discovered pathogenic *Rickettsia* spp. were first identified in arthropods and much later were reported in human cases, such as *R. parkeri* and *R. slovaca*. The former was identified in *Amblyomma maculatum* in 1937, and it was not until 60 years later that the first human case of *R. parkeri* infection was described [14]; the latter was described in *Dermacentor marginatus* in 1968, but the first documented case was reported several years later [1]. Recently, with the development of molecular techniques, new *Rickettsia* spp. have been identified in places where no rickettsioses had been reported, and some of these have been recognized as human pathogens [15]. The novel identified rickettsial species have enriched our understanding of rickettsioses. The objective of this study was to identify the rickettsial species in ticks collected from a specific area of Qing-Tibetan Plateau that had not been previously described for rickettsioses.

## Methods

### Study site and tick collection

The study site is located on the northeast edge of the Qing-Tibetan Plateau with an important forest zone and the main pasturing area in Gannan Tibetan Autonomous Prefecture (33°06“~ 36°10”N, 100°46“~ 104°44”E) that relies heavily on sheep, goat and yak farming for protein and local economy. Its average altitude is over 3,000 m. The annual average temperature here is 3.1 °C and annual precipitation is 582.7 mm. A total of 1,583 questing ticks were collected monthly between March and May 2011 by flagging the undergrowth with a flannel cloth. All ticks were identified as *Haemaphysalis qinghaiensis* based on the taxonomic key and morphological criteria [16].

### DNA extraction

Tick larvae and nymphs were pooled before DNA extraction; each tick pool consisted of 10 larvae or 5 nymphs, or a single adult tick. DNA was extracted by using the Puregene DNA purification kit (Qiagen, Beijing, China) according to the protocols.

### PCR reactions

The extracted DNA was examined for the presence of *Rickettsia* spp. *gltA* gene by using RpCS.409d and RpCS.1258n primers (5′-CCTATGGCTATTATGCTTGC-3′; 5′-ATTGCAAAAAGTACAGTGAACA-3′) and amplified a 770-bp fragments as described by Roux *et al.* [17]. Each positive sample was amplified with SFG rickettsiae specific primers Rr190.70 and Rr190.701 (5′-ATGGCGAATATTTCTCCAAAA-3′; 5′-GTTCCGTTAATGGCAGCATCT-3′) based on *ompA* gene and amplified at 617-680-bp fragments [18]. The reaction was performed in an automatic thermocycler (Bio-Rad, Hercules, USA) with a total volume of 25 μL containing 2.5 μL of 10 × PCR buffer (Mg^2+^ Plus), 2.0 μL of each dNTP at 2.5 mM, 1.25 U of Taq DNA polymerase (TaKaRa, Dalian China), 2.0 μL of template DNA, 1.0 μL of each primer (10 pmol), and 16.25 μL of distilled water. Positive and negative controls were included in each run. Cycling conditions for *gltA* and *ompA* amplification were: 4 min of denaturation at 94 °C, 35 cycles at 94 °C for 30 s, annealing for 30 s at 55 °C, and 72 °C for 45 s, with a final extension step at 72 °C for 10 min. PCR products were visualized by UV transillumination in a 1.0 % agarose gel following electrophoresis and staining with ethidium bromide.

### Sequencing and phylogenetic analyses

The PCR products of the partial *gltA* and *ompA* gene were purified using the TaKaRa Agarose Gel DNA purification Kit Ver.2.0 (TaKaRa, Dalian, China), ligated into pGEM-T Easy vector (Promega, USA), and transformed into *Escherichia coli* JM109 competent cells. Two recombinant clones were selected for sequencing using BigDye Terminator Mix (Sangon, Shanghai, China). The sequences obtained in this study were deposited in the GenBank (not including identical sequences) under accession nos. KT921891-KT921896. Sequences were analyzed by a BLASTn search in GenBank or by using the Clustal W method in the MegAlign software (DNAStar, Madison, WI). Phylogenetic trees were then based on the sequence distance method using the neighbor-joining (NJ) algorithm with the Kimura two-parameter model of the Mega 4.0 Software [19].

### Statistical analysis

The infection rates of *Rickettsia* infection in *H. qinghaiensis* ticks of larval and nymphal stages were estimated using the minimum infection rate [MIR, (the number of positive pools/the total number of ticks tested) × 100 %]. The results were analyzed using a Chi-square test in Predictive for Analytics Software (PASW) Statistics 18. A difference was considered statistically significant at *P* < 0.05.

### Ethical approval

This study was approved by the Animal Ethics Committee of Lanzhou Veterinary Research Institute, Chinese Academy of Agricultural Sciences.

## Results

The DNA of *Rickettsia* spp. was found in 179 (18.5 %) *H. qinghaiensis* tick pools by using PCR and primers specific for the *gltA* gene (Table 1). The infection rate of *Rickettsia* spp. was 14.7 % for adult *H. qinghaiensis* ticks. The MIRs were 5.9 % and 10.2 % for the larva and nymphs, respectively. Out of all tick specimens that tested positive for *Rickettsia*, 157 (16.3 %) tick pools were positive by PCR for the primer set Rr190.70 and Rr190.701 for the SFG rickettsiae *ompA* gene. The infection rate of SFG rickettsiae was 13.0 % for adult *H. qinghaiensis* ticks. The MIRs were 4.3 % and 9.8 % for the larva and nymphs, respectively. Twenty-two (2.3 %) tick pools that tested positive for the genus *Rickettsia gltA* gene were negative for SFG rickettsiae by using *ompA* primers. The infection rates of SFG rickettsiae were comparable in female and male ticks (13.2 % versus 12.7 %, *P* > 0.05).

**Table 1:** Prevalence of *Rickettsia* spp. in *Haemaphysalis qinghaiensis* ticks

Tick life stage	No. of ticks	Pool sizes	No. of pools	No. (%) of tick pools positive for *gltA*	No. of tick pools positive for *ompA*/no. examined/(%)^a^	No. (%) of tick pools positive for *gltA* and negative for *ompA*
Larva	460	10	46	27/(58.7)	20/27/(43.5)	7/(15.2)
Nymph	295	5	59	30/(50.8)	29/30/(49.2)	1/(1.7)
Female	506	1	506	80/(15.8)	67/80/(13.2)	13/(2.7)
Male	322	1	322	42/(13.0)	41/42/(12.7)	1/(0.3)
Total	1583	-	966	179/(18.5)	157/179/(16.3)	22/(2.3)

N = number of tested ticks; (%) - prevalence of infection

^a^Only ticks positive for the *gltA* gene were tested for the SFGR *ompA* gene

Seventy-one sequences (36 for *gltA* and 35 for *ompA*) were obtained in this study. Nucleotide sequence identities ranged from 97.0 % to 98.4 % for the *gltA* gene and from 87.8 % to 96.7 % for the *ompA* gene. After BLAST and CLUSTAL W alignment, 36 sequences of the *gltA* gene fragment (770 bp) were classified into three sequence types (ST), representing three different *Rickettsia* spp. isolates. ST1-3 had the highest nucleotide sequence similarities (99.0 %, 97.7 % and 97.4 %) to *R. raoultii* (GenBank accession no. KT261764). PCR amplification and sequencing showed the target fragments of the *ompA* gene were 617, 680 and 641 base pair for *Rickettsia* ST1-3, respectively. The sequence similarity of the *ompA* gene sequences of ST1-3 with that of *R. raoultii* was 79.4 %, 81.1 % and 81.4 %, respectively, which did not meet the threshold for *R. raoultii* [20].

**Fig. 1:**
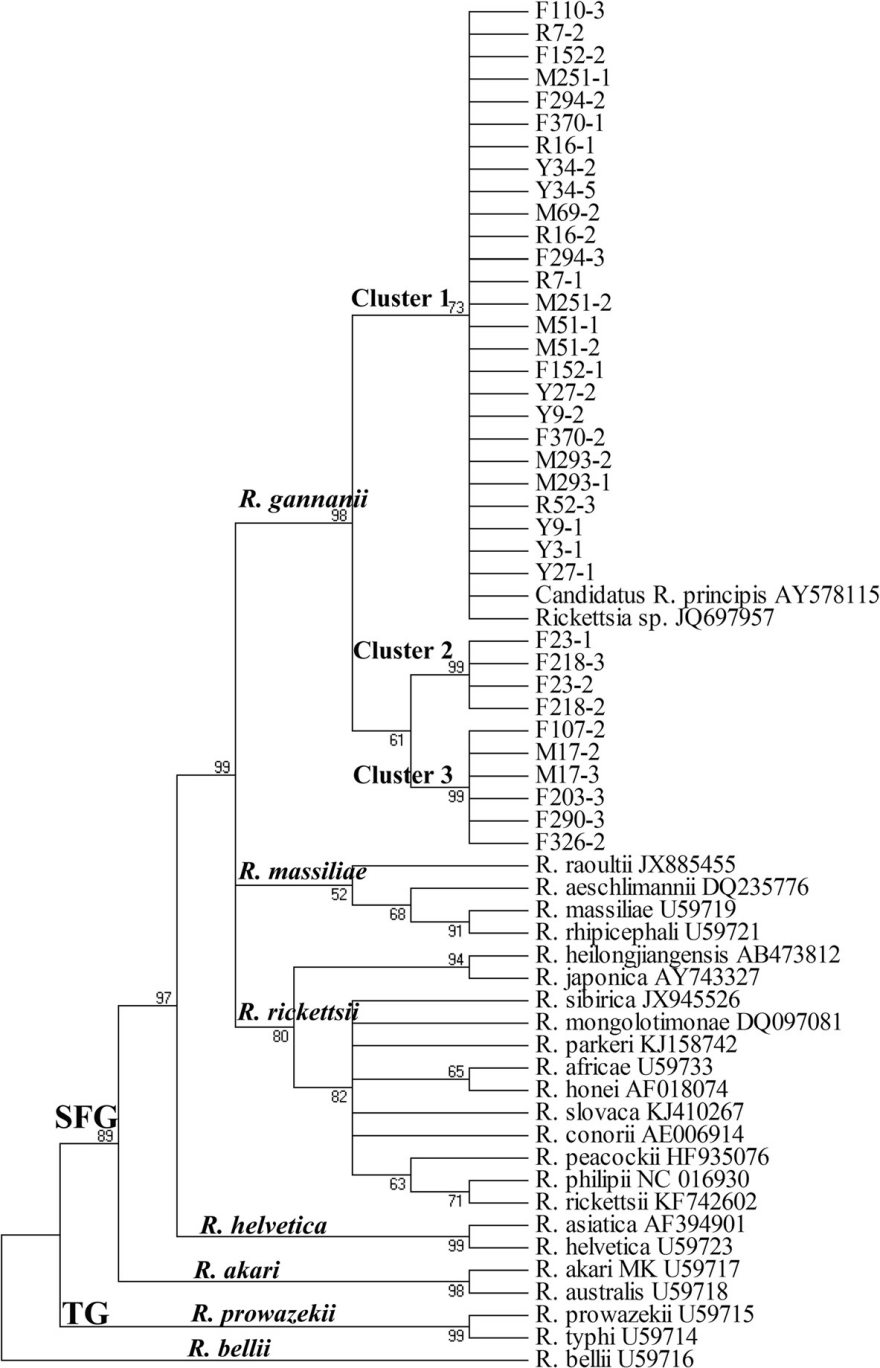
Phylogenetic tree based on *gltA* gene sequences of SFG rickettsiae. Bootstraps analysis was performed with 1000 replicates

**Fig. 2:**
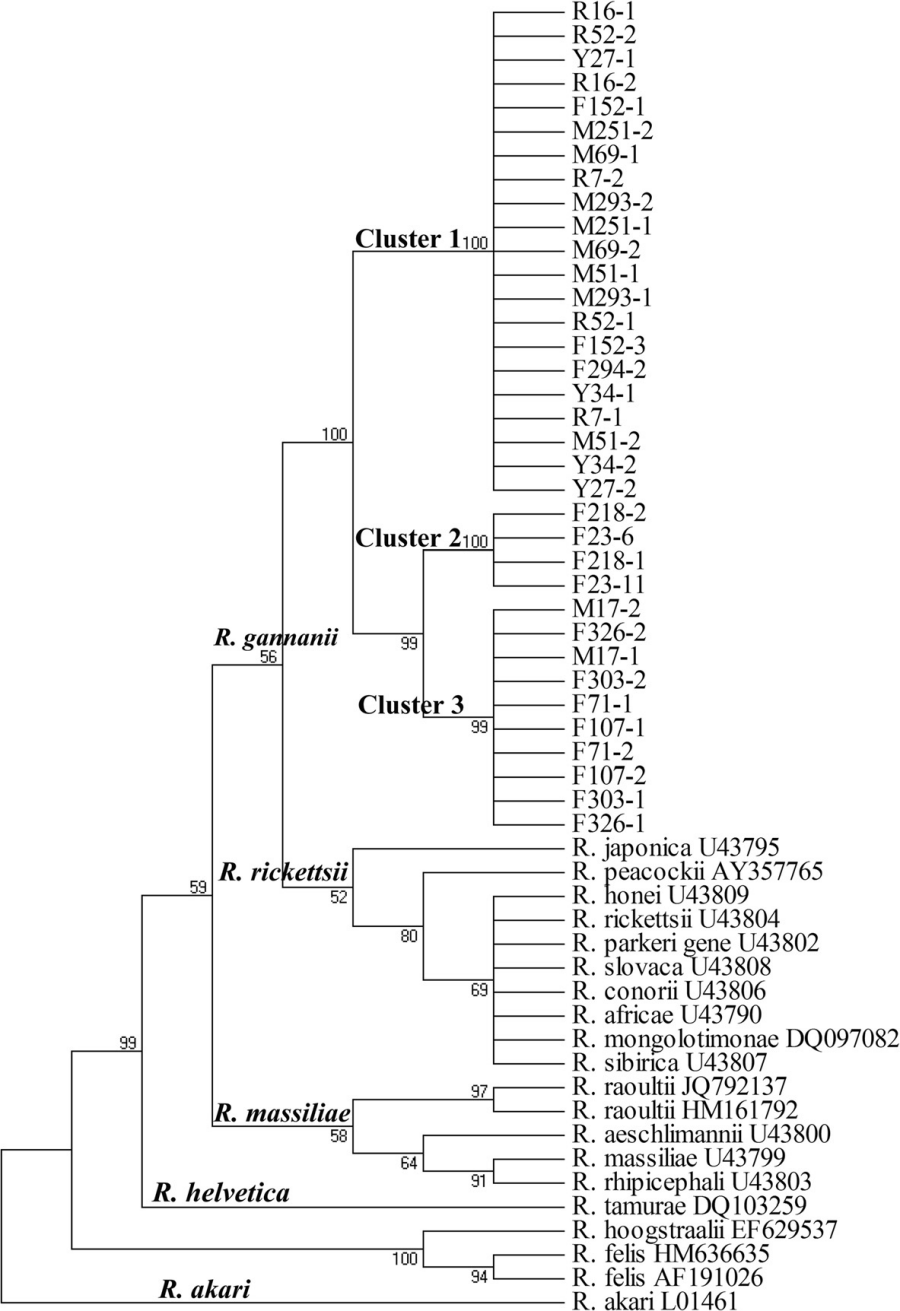
Phylogenetic tree based on *ompA* gene sequences of SFG rickettsiae. Bootstraps analysis was performed with 1000 replicates

Phylogenetic analyses revealed that the *gltA* gene sequences obtained in this study formed three distinct and well-defined clades inside the SFG rickettsiae (Fig. 1). Cluster one (ST1: Y27-1, Y34-5, etc.) was closely related to and clustered within the same clade as *Rickettsia* sp. strain “Tick-201-Mie-Hfla” isolated from *H. flava* found in Japan and the *Candidatus Rickettsia principis* isolated from *H. japonica* in Russia (GenBank accession no. JQ697957 and AY578115) [21]. Cluster two (ST2: F23-1, F23-2, F218-2 and F218-3) and three (ST3: F203-3, F290-3, M17-2, M17-3, F107-2 and F326-2) clustered independently from all known SFG rickettsiae sequences available on GenBank (Fig. 1). All SFG rickettsiae strains identified in *H. qinghaiensis* ticks were clustered together in a subgroup, which represents a sister taxon to and separates from other known four SFG rickettsiae subgroups (Fig. 1). Similar phylogenetic organizations were inferred from the sequence analysis of *ompA* gene (Fig. 2).

## Discussion

*Rickettsia* spp. have been recognized as emerging or re-emerging pathogens of public health relevance [2]. With the completion of the complete genome sequences, new perspectives on rickettsial evolution have been acquired. Apart from the traditional classification based on their morphological, antigenic, and metabolic characteristics, phylogenomic studies showed that the genus *Rickettsia* was classified into four different groups, including the well-defined SFG and TG, the *R. belli* group and the *R. canadensis* group [2, 22]. Until now, 27 characterized and dozens of as yet uncharacterized strains had been recognized worldwide [2]. The availability of specific and sensitive molecular tools used for taxonomic purposes have allowed for the identification of new species of *Rickettsia* in places where no rickettsioses had been reported. In the current study, the prevalence and molecular characterization of *Rickettsia* spp. was determined in *H. qinghaiensis* ticks, which was the dominant tick species and mainly recorded in northwestern China [23, 24].

In China, several *Rickettsia* spp. had been identified in ticks from different geographical locations, i.e. *R. sibirica* subsp. *sibirica* in *D. silvarum* and *D. sinicus* in northern China [8]; *R. sibirica* subsp. *mongolitimonae* in *H. asiaticum* in Inner Mongolia [25]; *R. heilongjiangensis* in *D. silvarum* in Heilongjiang and Yunnan [26, 27]; *R. japonica* in *H. longicornis* in Zhejiang [3]; *R. slovaca* and *R. raoultii* in *D. silvarum* in Xinjiang [28]; *R. monacensis* in *I. persulcatus* in central China [29]. Those studies indicate that numerous tick species maintain or transmit *Rickettsia*. In this study, we first reported the *Rickettsia* infections in *H. qinghaiensis* ticks, and high prevalences of *Rickettsia* (18.5 %) and SFG rickettsiae (16.3 %) infections were observed in study site. The hard ticks *H. qinghaiensis*, a distinctive species common in the Qing-Tibetan Plateau, is a three-host tick and preferentially infests domestic animals, such as sheep, goats, yaks and cattle [16, 23, 24]. Previous studies have demonstrated that *H. qinghaiensis* could transmit *Theileria* sp. and *Babesia* sp. for small ruminants [23, 24]. Recently, it has also been shown to be naturally infected with *Borrelia burgdorferi* (*s.l.*) and *Anaplasma phagocytophilum*, which are well-known human pathogens in the world [30, 31]. Taken together, our results suggest the potential role of *H. qinghaiensis* ticks as a reservoir host and carrier for piroplasmosis, Lyme disease, anaplasmosis and rickettsioses in this region.

The *gltA* and *ompA* genes were considered sufficiently variable to determine more reliable phylogenetic relationships within the genus *Rickettsia* [17, 20]. Previous report have demonstrated that SFG rickettsiae could be subdivided into four subgroups: *R. rickettsii*, *R. massiliae*, *R. helvetica* and *R. akari* [4]. In this study, phylogenetic tree inferred from *gltA* and *ompA* gene sequences revealed that three clusters of SFG rickettsiae were identified in *H. qinghaiensis* and these three different SFG rickettsiae clustered together in a subgroup separate from other known four SFG rickettsiae subgroups (Figs. 1 and 2). on the data presented here, we believe that the subgroup should be strongly considered as a new SFG rickettsiae subgroup, and we formally propose that these be named “*Candidatus* Rickettsia gannanii” related strains in recognition of the area where it was detected. In this subgroup, the cluster one may be the most dominant SFG rickettsiae distributed in study site (Figs. 1 and 2).

Some rickettsiae have been reported to be specifically associated with tick species, which are highly dependent on their biotopes [2]. Previous reports showed that *R. conorii conorii* seems to associate mainly with *Rhipicephalus sanguineus* sensu lato, *Haemaphysalis leachi* and *Rhipicephalus simus* in the Mediterranean region and Sub-Saharan Africa [2, 32]. In this study, three different SFG rickettsiae were identified in *H. qinghaiensis* ticks. The geographical distribution and associations between the “*Candidatus* Rickettsia gannanii” related strains and arthropod species should be further investigated.

In summary, our results showed discrepant SFG rickettsiae were circulating in northwest China. While the SFG rickettsiae identified in the *H. qinghaiensis* ticks have not been linked to human cases in the area, additional studies are therefore needed to determine if these *Rickettsia* spp. has any public health significance.

## Conclusions

This study represents the first published record of the detection of *Rickettsia* spp. in *H. qinghaiensis* ticks. Furthermore, on the basis of the sequence and phylogenetic data, it represents a putative novel SFG rickettsiae subgroup in *H. qinghaiensis* ticks from northwest China.
